# Glycogen storage diseases with liver involvement: a literature review of GSD type 0, IV, VI, IX and XI

**DOI:** 10.1186/s13023-022-02387-6

**Published:** 2022-06-20

**Authors:** Miriam Massese, Francesco Tagliaferri, Carlo Dionisi-Vici, Arianna Maiorana

**Affiliations:** 1 Division of Metabolism, Department of Pediatric Subspecialties, Ospedale Pediatrico Bambino Gesù, IRCCS, Piazza S. Onofrio 4, 00165 Rome, Italy; 2 Center for Rare Diseases and Birth Defects, Department of Woman and Child Health and Public Health, Fondazione Policlinico Universitario A. Gemelli IRCCS, Rome, Italy; 3 SCDU of Pediatrics, Azienda Ospedaliero-Universitaria Maggiore Della Carità, University of Piemonte Orientale, Novara, Italy

**Keywords:** GSDs, Glycogen storage diseases, Hypoglycemia

## Abstract

**Background:**

Glycogen storage diseases (GSDs) with liver involvement are classified into types 0, I, III, IV, VI, IX and XI, depending on the affected enzyme. Hypoglycemia and hepatomegaly are hallmarks of disease, but muscular and renal tubular involvement, dyslipidemia and osteopenia can develop. Considering the paucity of literature available, herein we provide a narrative review of these latter forms of GSDs.

**Main body:**

Diagnosis is based on clinical manifestations and laboratory test results, but molecular analysis is often necessary to distinguish the various forms, whose presentation can be similar. Compared to GSD type I and III, which are characterized by a more severe impact on metabolic and glycemic homeostasis, GSD type 0, VI, IX and XI are usually known to be responsive to the nutritional treatment for achieving a balanced metabolic homeostasis in the pediatric age. However, some patients can exhibit a more severe phenotype and an important progression of the liver and muscular disease. The effects of dietary adjustments in GSD type IV are encouraging, but data are limited.

**Conclusions:**

Early diagnosis allows a good metabolic control, with improvement of quality of life and prognosis, therefore we underline the importance of building a proper knowledge among physicians about these rare conditions. Regular monitoring is necessary to restrain disease progression and complications.

## Background

**Fig. 1:**
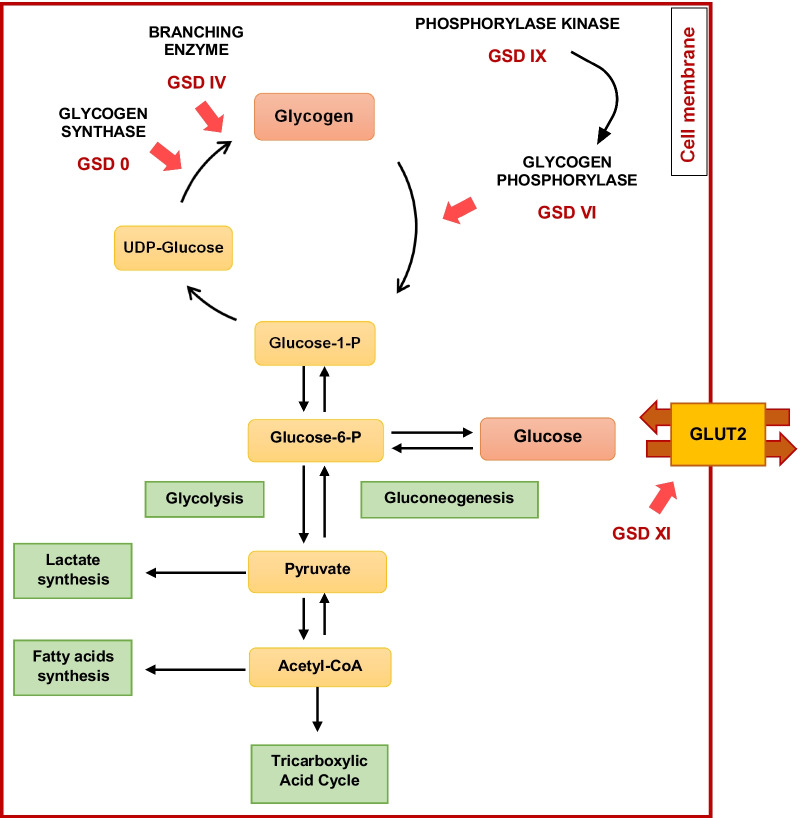
Simplified chart of glycogen metabolism in hepatocytes. After a meal, plasma glucose is metabolized either to pyruvate or stored as glycogen. Enzymes involved in GSDs type 0, IV, VI, IX and XI are pointed out. UDP glucose: uridine diphosphate glucose; glucose-1-P: glucose 1-phosphate; glucose-6-P: glucose-6-phosphate

Glycogen storage diseases (GSDs) are a group of rare inborn disorders of glycogen metabolism [1], a multibranched polysaccharide of glucose that serves as a rapidly consumable form of energy storage in mammalians. Glycogen is composed by chains formed by glucose units linked together linearly by α(1 → 4) glycosidic bonds from one glucose to the next, meanwhile branches are linked to the chains by α(1 → 6) glycosidic bonds [2]. After a meal, plasma glucose levels increase; then glucose is metabolized either to pyruvate or stored as glycogen (Fig. 1). In the first case, under aerobic conditions, pyruvate is either converted into Acetyl coenzyme A which enters the Krebs Cycle or is used to produce fatty acids. By contrast, under anaerobic conditions, lactate is derived from pyruvate as an alternative energy source. Glycogen synthesis and breakdown are regulated by different hormones such as glucagon, adrenaline, cortisol and insulin [1, 2].

GSDs may show a similar clinical presentation, although they may have a variable expressivity. Their overall incidence is approximately 1:10,000 live births [3] and they are classified, depending on the defective enzyme and the primarily affected tissues [1]. GSDs types 0a (gene GYS2, OMIM # 240600), IV or Andersen disease (GBE1, OMIM # 232500), VI or Hers disease (PYGL, OMIM # 232700), IXa (PHKA2, OMIM # 306000), IXb (PHKB, OMIM # 261750), IXc (PHKG2, OMIM # 613027) and XI or Fanconi-Bickel syndrome (SLC2A2, OMIM # 227810) are included into the group of hepatic GSDs together with types I and III. Differently from the two latter GSDs, the former types have been considered as more benign, however there are growing evidences of a significant clinical variability, with some patients exhibiting a severe phenotype [4]. All GSDs are autosomal recessive except for type IXa, which shows an X-linked inheritance [3]. Hepatic GSDs share some clinical signs, such as fasting hypoglycemia and hepatomegaly [1, 3], albeit GSD type 0 usually shows hypoglycemia without signs of liver involvement [5]. Furthermore, some GSD type IXa may present merely with ketotic hypoglycemia [6]. Patients with GSDs types 0, IV, VI, IX and XI are usually good at birth, with symptoms developing in early infancy or childhood. Except for a few types, the prognosis is good if a strict dietary treatment and surveillance of complications are achieved. Although a clear genotype–phenotype correlation has not been drawn, different mutations may justify the wide spectrum of phenotypes, with different grade of severity [7]. In the recent years, additional knowledge has been gained about the pathophysiology, clinical course and treatment of these conditions and several case series are now available. In the present review we provide an overview about clinical and laboratory characteristics of GSDs types 0, IV, VI, IX and XI, in order to support a punctual diagnosis, which can influence the prognosis and the quality of life of the patients, and to highlight the importance of a regular follow-up of the disease progression leading to the organ damage.

## Methods

The literature search occurred in November 2021. All potentially relevant articles were selected from three electronic databases: Google Scholar, Medline and PubMed. Search terms such as “glycogen storage disease”, “glycogen storage disease type 0”, “GYS2 defects”, “glycogen storage disease type IV”, “GBE1”, “glycogen storage disease type VI”, “PYGL”, “glycogen storage disease type IX”, “PHKA2”, “PHKB”, “PHKG2”, “glycogen storage disease type XI”, “GLUT2 deficiency”, “Fanconi-Bickel syndrome” were used in various combinations and permutations.

## Results

### Overview

#### GSD type 0

GSD type 0a is caused by defects of the hepatic isoform of glycogen synthase, encoded by *GYS2* (chromosome 12p12.2), which catalyzes the linear addition of glucose residues to the branching structure of glycogen. Mutations of the muscle-specific isoform of glycogen synthase encoded by *GYS1* gene originate GSD type 0b. Clinically, it exhibits weakness, exercise intolerance and arrythmias without liver involvement [8].

A focus on GSD subtype 0a is provided in this review and it will be referred to as “GSD type 0”. The onset of symptoms occurs before 3.5 years old on average [9]. Affected toddlers are incapable of synthetizing glycogen and having adequate glycemic response to stress or fasting periods. For this reason, they show fasting ketotic hypoglycemia, that can be either symptomatic or asymptomatic. Symptomatic hypoglycemia is revealed by pallor, sweating, hyporeactivity, lethargic state until generalized seizures [10, 11]. After a meal or an oral glucose tolerance test (OGTT), hyperglycemia and hyperlactatemia are observed [12], whereas hypoglycemia can manifest after three hours from the last feeding [13]. Patients cannot switch to gluconeogenesis rapidly enough to ensure a normal hepatic glucose output. It could be speculated that the post-prandial hyperglycemia typical of GSD type 0 suppresses the glucagon activity and that glucagon to insulin ratio remains too low to stimulate phosphoenolpyruvate carboxykinase, the rate-limiting enzyme for gluconeogenesis [12]. Fasting glucagon stimulus test may not increase glycemia as the expected, confirming the presence of poor glycogen deposits [14], with a response in the fed state not consistent among patients [10]. The presentation with post-prandial hyperglycemia and glycosuria, along with fasting ketonuria, places GSD type 0 in differential diagnosis with early phases of diabetes mellitus and Fanconi-Bickel syndrome [14]. However, ketonuria is not a constant finding and measurement of ketones in blood is preferable [13]. Hepatomegaly is not associated to this disorder [10, 12, 15–17], although a mild liver enlargement has been described in some patients [13]. Slightly different phenotypes might be related to different degrees of enzyme residual activity. Growth failure is reported [9, 12]. Furthermore, two patients lacking of post-prandial hyperglycemia/hyperlactatemia were diagnosed with GSD type 0 by targeted NGS [18].

#### GSD type IV (Andersen disease)

The branching enzyme deficiency (*GBE1* gene, chromosome 3p14) causes the GSD type IV or Andersen disease. The branching enzyme adds a segment of a minimum of six α-1,4 linked glycosyl units into an α-1,6 position; this activity is fundamental for the correct glycogen storage and degradation. Indeed, GBE1 deficiency induces the accumulation of an amylopectin-like molecule called polyglucosan, an insoluble polymer which has fewer branching points and longer outer chains than normal glycogen [19].

GSD type IV includes two hepatic subtypes (the classical progressive and the non-progressive hepatic disease), three neuromuscular subtypes (distinguished in the perinatal, congenital and childhood disease, depending on the age of onset) and a multisystem form (adult polyglucosan body disease). Indeed, the GSD type IV has a very heterogeneous clinical presentation, ranging between myopathy, cardiomyopathy, neuropathy and liver failure variously combined [20, 21]. One of the first reports available in the literature described this condition as “a new cause of floppy infant” [22].

The classical progressive hepatic form is characterized by an initially normal phenotype with rapid deterioration in infancy, when hepatomegaly and failure to thrive appear. Prolonged partial thromboplastin time and prothrombin time progressively develop, along with hypoalbuminemia. Linear glycogen molecules can be metabolized, so this prevents severe hypoglycemia. Nevertheless, it may develop in the final stages of liver failure [23]. The final evolution is toward progressive fibrosis, cirrhosis and its complications, such as portal hypertension, ascites and esophageal varices, with death occurring before the age of five owing to liver failure [24, 25]. Liver transplantation remains the only effective treatment for patients with the progressive hepatic subtype of GSD type IV who develop liver failure. However, some patients treated by liver transplantation displayed over time extrahepatic manifestations, such as cardiomyopathy and myopathy [26–29].

The non-progressive hepatic disease exhibits different grades of severity, with the milder phenotype showing hepatomegaly and inconstant elevation of transaminases. McConkie-Rosell et al. [30] in 1996 described four patients with hepatomegaly associated to elevated hepatic enzymes at young ages, who showed liver fibrosis without signs of progression toward cirrhosis nor portal hypertension nor liver failure in a follow-up ranging from 2 to 19 years. Conversely, Dhawan et al. [31] described a patient who only showed hepatomegaly until 12 years old, when he rapidly developed cirrhosis with portal hypertension requiring liver transplantation. The severity of the phenotype might depend both on the involvement of different tissue isozymes [1] and the residual activity of branching enzyme [21]. GSD type IV patients exhibit a continuum of different phenotypes, with extremely variable clinical features. Hypoglycemia has traditionally been considered a late manifestation, related to hepatocellular dysfunction; notably, a recent report documented fasting intolerance in patients without any sign of liver involvement [26].

The neuromuscular variants of GSD type IV are very rare; nevertheless, the perinatal variant should be considered one of the differential diagnoses in neonates with severe hypotonia and in pregnancies complicated by polyhydramnios, fetal hydrops, reduced fetal movements, arthrogryposis, hypoplastic lungs of unknown etiology [19–21, 32]. The congenital neuromuscular subtype begins in the newborn period with profound hypotonia, respiratory distress, and dilated cardiomyopathy and, as well as the previous one, results in death in the neonatal period. The childhood neuromuscular subtype, which is the rarest one, has most variable course. Its onset ranges from the second life decade with a mild disease course to a more severe, progressive course resulting in death in the third decade [21]. Neurological adult form can present as isolated myopathy or as widespread upper and lower motor neuron lesions (adult polyglucosan body disease), which presents usually after the age of 50 years. Its hallmarks are progressive spastic paraparesis, neurogenic bladder, and axonal neuropathy [29, 33].

#### GSD type VI (Hers disease)

*PYGL* gene (chromosome 14q21-q22) mutations are associated to GSD type VI, also known as Hers disease [34]. Affected individuals lack the glycogen phosphorylase activity, which breaks up glycogen into glucose units as a response to hypoglycemia. This is a rate limiting step in glycogen degradation, hence the untreated child shows moderate fasting hypoglycemia with mild ketosis, hyperlipidemia, elevated liver enzymes, abdominal distension, hepatomegaly and growth failure. Pre-albumin is also reduced [34, 35]. Symptoms begin at the pre-school age; hypoglycemia may originate during prolonged fasting, illnesses or stressful conditions, thus a strict surveillance must be realized in these cases [34, 36]. Ketotic hypoglycemia without hepatomegaly has also been recently described in GSD type VI as the only sign of disease [6].

GSD type VI was previously considered as a mild disease. However, recent reports highlighted the possibility of a progression to fibrosis and cirrhosis, and a degeneration to hepatocellular carcinoma, so a rigorous long-term monitoring of hepatic function is needed [4, 37, 38].

#### GSD type IX

The above mentioned glycogen phosphorylase is functionally activated by the phosphorylase kinase (PhK), a serine/threonine-specific protein kinase. PhK is composed of four different subunits: α, β, γ and δ. Subunits α and β have regulatory functions, the γ-subunit has catalytic function and δ-subunit is a calmodulin protein. The subunits possess tissue-specific isoforms; the liver-specific isoforms of the α-, β- and γ-subunits are encoded by *PHKA2*, *PHKB* and *PHKG2* respectively, and are causative of GSD IX subtypes IXa, IXb and IXc [39]. However, *PHKB* is expressed in both liver and muscle. Furthermore, the α- and γ-subunits have a muscle-specific isoform, encoded by *PHKA1* and *PHKG1* respectively. The α-subunit isoforms are inherited in an X-linked fashion, while the other isoforms have an autosomal recessive inheritance [40]. Furthermore, the subtype IXa is distinguished in types XLG I and XLG II, two clinically similar entities basically differing for the possibility to discover the enzyme deficiency on erythrocytes [41].

GSD type IX represents the most frequent type of glycogen storage disease, with a prevalence of 1:100,000 births [42]. The phenotype is dominated by short stature, a characteristic round face (“doll face”), liver enlargement with elevated transaminases, triglycerides and cholesterol, normal uric acid and lactic acid concentrations. Renal tubulopathy is an inconstant finding [37]. Hypoglycemia is not always pronounced because gluconeogenesis and fatty acid oxidation are intact, and normal blood glucose concentrations are often maintained [43, 44]. When hypoglycemia is present, ketosis is associated in fasting conditions [45]. More recently, isolated ketotic hypoglycemia without hepatomegaly has been related to PhK deficiency, mostly due to *PHKA2* mutations [6].

PhK deficiency was generally considered a benign condition, with symptoms of hypoglycemia, hepatomegaly and growth retardation improving after the early introduction of a strict dietary treatment [43]. Nevertheless, recent studies have focused on the existence of complications and different prognosis depending on the causative mutation. For instance, an evolution to liver fibrosis and chronic liver disease associated to *PHKA2* mutations has been reported [46, 47]. Furthermore, Burwinkel et al. [47] reported two patients displaying peculiar features such as kidney dysfunction due to renal tubular acidosis and neurological involvement with outcome of delayed cognitive and speech abilities, confirming that the spectrum of the disease is extremely broad.

The phenotype associated to *PHKB* mutations is similar to what observed in the milder *PHKA2* mutations; symptoms related to muscular involvement may be present [48]. *PHKG2* associated phenotypes show a more severe presentation [49]. The clinical spectrum includes fasting hypoglycemia, hepatomegaly, elevated transaminases, liver fibrosis, cirrhosis, muscle weakness, hypotonia, motor developmental delay, growth retardation and fatigue [50, 51]. More recently, the presence of variable degree of liver fibrosis and cirrhosis still in early childhood has been reported in the three subtypes [4].

#### GSD type XI (Fanconi–Bickel syndrome)

GSD type XI is caused by defective glucose and galactose transporter GLUT2 (*SLC2A2* gene, chromosome 3q26.1–26.3), expressed in hepatocytes, pancreatic β-cells, enterocytes and renal tubular cells [52]. This condition causes impaired influx and efflux of glucose from the aforementioned cell types, and it may have a role in insulin secretion [53]. This is a severe disease with a peculiar clinical presentation. Both transport and metabolism of glucose and galactose are defective, with subsequent increased hepatorenal glycogen storage leading to hepatomegaly. At early stages, a slight elevation of transaminases is recorded.

Neonatal screening may show hypergalactosemia but cataract is not present in this condition [54]. Impaired renal glucose reabsorption, as well as the accumulation of glucose in the liver, which reduces glycogen breakdown, causes fasting ketotic hypoglycemia. Conversely, in the fed state hyperglycemia is observed. This may be due to the impairment of glucose transportation from the enterocytes and decreased glucose uptake by the liver, consequent to impaired insulin secretion [53]. Signs and symptoms usually begin between 3 and 10 months of life with poor feeding, failure to thrive and laboratory findings as glycosuria [54]. The proximal renal tubular dysfunction implicates glycosuria, proteinuria, phosphaturia, aminoaciduria and bicarbonate wasting, resulting in a metabolic hyperchloremic acidosis with normal anion gap [52]. Hypercalciuria is a constant finding. In older children, pubertal delay and hypophosphatemic rickets are described [55, 56]. Tendency to hyponatremia and hypokalemia is frequent owing to renal losses. Polyuria may be present as a consequence of high osmotic load. Patients may exhibit chronic diarrhea secondary to intestinal malabsorption. Hyperlipidemia is recorded, leading to moon-shaped face and fat deposition on shoulders and abdomen, which are typical features [1, 54]. Notably, a GLUT2 deleted mouse model exhibited an increased expression of ChREBP (Carbohydrate Response Element Binding Protein) which in turn activates the lipogenic target genes transcription [57]. Patients with Fanconi-Bickel syndrome manifest a dysregulation of glucose homeostasis, with presentation of fasting hypoglycemia, post-prandial hyperglycemia, glucose intolerance, transient neonatal diabetes, gestational diabetes and frank diabetes mellitus. Impaired glucose control along with low birth weight suggest that GLUT2 might have a role in insulin physiology from fetal to adult age [53]. A few cases of patients with a similar phenotype but without any mutation of SLC2A2 were reported, suggesting other genes involved in the pathogenesis of this condition, remaining unknown so far [58]. Remarkably, the mutations p.R63W and LRG_483t1:c.427-1G > A in the *HNF4α* gene cause hyperinsulinemic hypoglycemia associated to hepatomegaly and renal Fanconi syndrome. These *HNF4α* mutations might decrease the SLC2A2 expression in both liver and kidney, resulting in nonfunctional GLUT2 and are responsive to therapy with diazoxide [59, 60].

### Focus on the main GSDs clinical features

#### Glucose homeostasis

Fasting ketotic hypoglycemia is a hallmark of hepatic GSDs [26, 61]. Patients with GSD type 0 and XI show also a typical post-prandial hyperglycemia [12, 16, 53]. In GSD type IV, hypoglycemia can appear late in the clinical course, but it can be also found in patients without signs of liver disease [26]. Ketotic hypoglycemia without hepatomegaly has also been recently described in GSD type VI and IX [6]. Futhermore, isolated ketonemia with normoglycemia has been described in patients with GSD types VI and IX [62]. GSD type XI exhibits a wide range of alterations in glucose homeostasis, including fasting hypoglycemia, hyperglycemia in the fed state, glucose intolerance up to diabetes mellitus in rare cases [53].

#### Lipid homeostasis

Elevated triglyceridemia and cholesterolemia are common findings in GSDs with liver involvement. In these conditions, the dysregulation of glucose metabolism leads to fasting intolerance, enhancing secondary lipolysis and increased mitochondrial fatty acid oxidation [1]. In GDS type XI, the administration of statins may be required [63]. In the other forms, the dyslipidemia is generally moderate and an appropriate nutritional therapy is effective to reduce plasma lipid values [64].

#### Liver involvement

As previously mentioned, the distinctive element of the glycogen synthase deficiency is the absence of hepatomegaly, since hepatic glycogen storage is impaired [9, 12], although enlarged liver has been reported in some cases of GSD type 0 [13, 16]. By contrast, hepatomegaly is the hallmark of the GSD type IV, VI, IX and XI with various degrees of severity, which may show an improvement after puberty in treated GSD type IX patients [64, 65]. However, a progression of the liver disease may occur despite a reduction of the liver size [44].

In GSD type IV the accumulation of abnormal glycogen, less soluble than normal glycogen, causes a foreign body reaction with consequent osmotic swelling and cell death [50], leading to interstitial fibrosis evolving toward cirrhosis [24]. Liver fibrosis is outlined also in individuals with GSD types VI [38, 66] and IX [4, 51]. Particularly, in GSD type IX fibrosis has been recently reported to range between 33 and 95% depending on the subtype still in early infancy [4].

Furthermore, cirrhosis has recently been depicted in GSD type VI [38]. Among the GSD IX subtypes, the progression to liver cirrhosis had initially been described only in patients affected by *PHKG2* mutations [49]. Nevertheless, Tsilianidis et al. [43] described precocious liver cirrhosis in two patients with *PHKA2* mutations. More recently, early appearance of liver cirrhosis in a 2 years old child with homozygous mutations in *PHKB* has been reported [40].

Tumor degeneration is described in GSD type IV, VI and IX. Hepatocellular adenoma and carcinoma have been described in GSD type IV [67]. GSD type VI can be rarely complicated by focal nodular hyperplasia [68] and one case of hepatocellular carcinoma has been reported to date [69]. With regards to GSD type IX, hepatocellular adenomas have been reported in IXa and IXb subtypes [4, 44]. Furthermore, the development of hepatocellular carcinoma associated to GSD type IXc has recently been described [70]. In GSD type XI, liver histology shows marked accumulation of glycogen in hepatocytes along with steatosis. The degeneration to hepatic adenomas or carcinomas is rare [54]. The first case of hepatocellular carcinoma in a young boy affected by Fanconi-Bickel syndrome was described in 2017 by Pogoriler and colleagues [71].

#### Renal involvement

Renal involvement is not described in GSDs types 0 and VI to date.

Conversely, individuals affected by *PHKA2* and *PHKG2* mutations can display renal tubular acidosis and tubulopathy with secondary development of rickets in a patient with GSD type IXc. The establishment of an adequate nutritional therapy improves tubular acidosis [37].

In addition, renal involvement represents a hallmark of GSD type XI, in which the renal epithelial cells are damaged by the accumulation of glycogen and monosaccharides; this alteration leads to proximal tubular dysfunction, documented by glycosuria and aminoaciduria. Although this condition is related to a severe phenotype, rare cases of patients with mild renal dysfunction have been described [8].

#### Growth impairment and bone metabolism

Normal length and weight at birth are usually observed, suggesting that the metabolic disorders do not interfere with fetal growth [11, 19, 72], except for newborns with GSD type XI, which are typically low birth weight [53].

Patients diagnosed with GSD type 0 may show either normal or poor growth with a delayed bone age in early childhood [15, 17]. A catch-up growth has been described after the introduction of adequate dietary therapy, comprising uncooked cornstarch [9]. Osteopenia is a possible complication [5].

Growth impairment is not a hallmark of GDS type IV and it may be present or not, depending on the causing mutation and the clinical subtype [24].

In contrast, short stature is a common feature in GSD types VI and IX, with a variability in the degree of improvement of parameters in treated patients reaching the adult age [36]. Most individuals affected by PhK deficiency achieve standard adult stature parameters, but they show a peculiar growth pattern, with an initial growth retardation in the first 2–3 years of age, followed by a gradual normalization of the linear growth [65]. Abnormal bone mineralization with and without osteopenia has been reported in GSDs types VI and IX [37, 73]. Dietary deficiencies and chronic ketosis are speculated to be contributory factors [37]. Rickets has been reported in a case of GSD type IXc, due to renal tubulopathy with an inappropriate parathyroid response [37].

Severe growth impairment is described in Fanconi-Bickel syndrome. Patients affected by proximal renal tubular dysfunction of variable genetic causes show growth retardation ascribed to renal losses but the short stature observed in Fanconi-Bickel syndrome is more pronounced, suggesting other mechanisms not clearly understood [74]. Newborns are generally low birth weight, likely effect of the insulin deregulation starting in utero [53]. Furthermore, dwarfism is a striking feature in adult patients [1], with scarce response to nutritional therapy. Remarkably, Pennisi and colleagues [63] reported a substantial improvement of height and weight by the administration of nocturnal enteral nutrition from the age of 1 year, in five patients. The nocturnal enteral feeding provided an appropriate glucose rate intake for age (8–9 mg/kg/min in infants, 5–7 mg/kg/min in children and 2–4 mg/kg/min in adolescents); diet granted 55–60% of caloric intake as carbohydrates, 30% as lipids, and 10% as proteins. Four patients were supplemented with uncooked cornstarch in the enteral nutrition. All patients showed a catch-up growth from 3rd percentile or below to 10th/50th percentiles for height and weight at last follow up (age range 15–24 years). Notably, untreated patients reached an adult height ranging from 131.5 to 153 cm [75].

Among all GSDs, bone is mostly affected in GSD type XI, where hypophosphatemic rickets, frequent fractures and bone deformities are described as a result of the renal tubular dysfunction [76]. Limbs deformities and lumbar hyperlordosis may appear in patients with delayed diagnosis, as observed in developing countries [74].

#### Muscular and cardiac involvement

Skeletal muscle and myocardial involvement is not observed in GSD type 0a [9].

Heart failure after orthotropic liver transplantation has been described in patients with the progressive liver form of GSD type IV with no previous history of cardiac involvement [27, 28]. This could be due to a progression of disease, despite liver transplantation. Indeed, in patients dead after liver transplantation, amylopectin deposits have been observed in different organs and tissues (myocardial fibers, skeletal muscle fibers, central and peripheral nervous system cells, macrophages) at autopsy [77]. A good clinical response to liver transplantation may be explained by a mechanism of microchimerism, through which the donor cells transfer the deficient enzyme to the host cells, thus reducing amylopectin deposits [78].

Mild to severe myopathy and dilated cardiomyopathy are also described in the neuromuscular forms of GSD type IV [24, 79]. Remarkably, cardiomyopathy has been reported as the sole presenting symptom of branching enzyme deficiency in one case [21].

Muscular cramps or fatigue after physical exercise have been recorded in a minority of reports of GSD type VI, usually related to undertreatment and protein deficiency [36]. Muscle weakness may or may not be observed in PhK deficiency with any genotype [48, 49].

In a recent case series, asymptomatic left ventricular and septal hypertrophy was reported in a patient with GSD type VI, and interventricular septal hypertrophy was found in a patient with GSD type IXb. The authors recommended echocardiogram every 1–2 years for patients with GSD type VI and IX after 5 years of age [44]. A systematic review of the literature did not reveal other individuals with GSD type VI or IX and cardiac problems [3]. Muscular involvement can be seen in the context of dyselectrolytemia in GSD type XI [52], revealed by exercise intolerance and rhabdomyolysis [33].

#### Psychomotor development and nervous system

Developmental delay has been described in 22% of children with GSD type 0 [5], whereas undiagnosed GSD type 0 was associated to a higher incidence of neurodevelopmental impairment caused by severe recurrent hypoglycemia. In these patients, hypoglycemia is often non symptomatic, as the loss of neuroglycopenic signs in recurrent hypoglycemia is notable [14]. The phenomenon, noted as hypoglycemia-associated autonomic failure, is due to a defective glucose counter-regulation with an attenuated sympathoadrenal and neural response leading to reduced neurogenic and cerebral symptoms [80]. Seizures are uncommon [5].

Mild developmental delay was also reported in GSD types VI, IX and XI [36, 76]. With regards to GSD type IX, a recently published literature review with data analysis of 174 patients outlined that a mild developmental delay was present in type IXc, with a frequency two times higher than other subtypes [4].

With respect to the motor impairment, GSD type IV, VI, IX and XI deserve to be mentioned.

In the progressive hepatic GSD type IV the muscle tone is often normal at the time of diagnosis, but progression to generalized hypotonia may develop within the two years of life [20]. GSD type IV shows a complex involvement of neuromuscular system. The perinatal and congenital neuromuscular subtypes show severe congenital hypotonia and respiratory distress, which impose the differential diagnosis with spinal muscular atrophy and the inherited storage disorders with neuromuscular involvement (eg Pompe disease, Zellweger disease) [19, 20]. Patients affected by the childhood neuromuscular subtype show skeletal myopathy and hypotonia and may experience motor developmental delay with possible death in early adulthood [24]. Furthermore, progressive spastic paraparesis, neurogenic bladder, and axonal neuropathy have been described in the adult polyglucosan body disease [33]. This is a rare condition due to the accumulation of polyglucosan bodies into the neuronal axons and processes of astrocytes and oligodendrocytes. This process leads to a sensorimotor neuropathy, with involvement of both upper and lower motor neuron and onset around the fifth decade. The clinical presentation is very variable, characterized by symptoms of neurogenic bladder, legs weakness, gait disturbances, spasticity, cognitive dementia with different grades of severity. Among the neurologic signs, spasticity, reduced ankle reflexes, extensor plantar response and sensory deficits of lower extremities are seen [81].

Mild hypotonia was reported in a few GSD type VI patients [36].

Hypotonia and motor delay can be rarely associated to *PHKB* and *PHKG2* mutations [48, 51]. With regards to *PHKA2* mutations, Lau et al. [82] and colleagues described a young patient exhibiting an impairment of gross motor ability in the context of a borderline developmental delay. Hypotonia and motor impairment were also recorded in GSD type XI [1, 3].

A summary of the main clinical features of the GSDs is provided in Table 1.

**Table 1:** Main clinical features of GSDs 0, IV, VI, IX and XI

	Liver			Renal tubulopathy	Growth retardation	Osteopenia	Muscle/heart involvement	Developmental delay/Neuropathy
	Hepatomegaly	Fibrosis/ cirrhosis	Hepatocellular carcinoma					
GSD 0 a	+/−	−	−	−	+/−	+/−	−	+/−
GSD IV	+/−	+/−	+/−	−	+/−	−	+/−	+/−
GSD VI	+/−	+/−	+/−	−	+	+/−	+/−	+/−
GSD IX a	+/−	+/−	−	+/−	+*	+/−	+/−	+/−
GSD IX b	+/−	+/−	−	−	+*	+/−	+/−	+/−
GSD IX c	+/−	+/−	+/−	+/−	+*	+/−^§^	+/−	+/−
GSD XI	+	−	+/−	+†	+	+^§^	+/−	+/−

^†^Fanconi-like renal tubular acidosis presenting with glycosuria, proteinuria, phosphaturia and aminoaciduria

*Growth is delayed but it generally improves, with subjects reaching normal adult height

^§^Hypophosphatemic rickets

### Diagnosis

A careful clinical history and examination together with laboratory findings may suggest the diagnosis. An OGTT can be realized when GSD types 0, VI and IX are suspected; in all forms elevated lactate will be recorded at 120 min. Patients with GSD type 0 will show hyperglycemia within the first two hours, then hypoglycemia might be observed at a prolonged OGTT, likely due to hyperglycemia-induced hyperinsulinemia [12]. In the past, enzymatic activity in peripheral blood cells and cultured skin fibroblasts was performed. The reduced activity of branching enzyme in leucocytes, erythrocytes and fibroblasts confirmed the diagnosis of GSD type IV, however normal activity in leukocytes could not exclude the neuromuscular forms [24]. In GSD type VI a reduced phosphorylase activity could be detected in erythrocytes and leukocytes [35]. The deficiency of phosphorylase kinase activity could be outlined in leucocytes, erythrocytes and fibroblasts, except for the forms associated to certain missense mutations of *PHKA2* and *PHKB* [41, 47]. In the case of normal enzymatic activity in peripheral blood cells, a liver biopsy for enzymatic assay in hepatocytes was assessed [47]. More recently, molecular analysis became the method of choice to confirm the diagnosis for each GSD type. However, these forms may have similar clinical and biochemical presentation. Thus, performing single gene analysis would result time consuming and expensive. In the last decade, next generation sequencing technology (as gene panel or clinical exome) found a wide application for the diagnosis of inborn errors of metabolism for the genetic heterogeneity of these conditions, allowing to carry out large molecular characterization of patients within an useful timeframe and at a reasonable cost [18]. However, with these techniques non-coding and structural variants cannot be captured, the gene coverage may be variable, deletions/duplications can be missed, and the identification of variants of uncertain significance poses a diagnostic challenge [83, 84]. In these cases, histology and enzyme testing on a liver biopsy specimen may be required to confirm the diagnosis [37].

### Treatment and follow-up

A strict dietary regimen high in proteins and low in simple carbohydrates, which includes frequent intake of complex carbohydrates such as maltodextrin and uncooked cornstarch, is fundamental to prevent hypoglycemia in ketotic GSDs [6]. Indeed, a metabolic imbalance results in overnight hypoglycemia and ketosis, that are associated to short stature, osteopenia, and neurologic complications [43]. GSDs types 0, VI and particularly type IX would benefit from a strict glycemia monitoring. A minority of patients with mutations of *PHKA2* and *PHKG2* associated to a severe phenotype often require overnight feeding to maintain euglycemia [85]. Since gluconeogenesis is preserved, protein supplementation provides gluconeogenic precursors that can be used for repletion of Krebs cycle intermediates and endogenous glucose production in GSD types 0, IV, VI and IX. By improving glucose homeostasis, hepatic glycogen accumulation and secondary complications might be restrained. Diet should be high in protein and provide 2–3 g of protein/kg or ~ 20–25% of total calories [26, 37, 61]. High protein intake is especially needed in GSD type VI to improve muscle function [44]. Carbohydrates should provide ~ 45–50% of total calories, with complex carbohydrates and protein in every meal. The dosage of raw cornstarch 1 g/kg at bedtime allows to maintain normoglycemia for 4–8 h in infants and children [37]. In 2015 Ross and co-workers [85] described the efficacy of an extended-release cornstarch (Glycosade) in GSD types 0, III, VI and IX to achieve a longer time of euglycemia during the night, with stable values of other markers of metabolic control and hepatic function. In the United States, the extended-release cornstarch preparation has been approved for nocturnal use in GSD patients above 5 years of age. However, the administration of Glycosade in patient between 2 and 5 years of age resulted safe and effective as well [86]. Adverse effects such as abdominal distension, diarrhea and flatulence have been reported, but to date they were not recorded in patients with GSD types 0, VI and IX [61].

Patients with GSD type 0 are treated with frequent feeds of hyperglucidic diet plus cornstarch and protein supplementation. Patients with GSD type IV are managed with hyperglucidic diet plus cornstarch, nocturnal enteral feeding, protein enrichment with the aim to limit the accumulation of glycogen, to prevent catabolism and to improve growth and fasting tolerance. The more severe forms are treated with liver transplantation [26]. For GSD type XI, Pennisi and co-workers [63] proposed the nocturnal enteral nutrition in younger children and in patients with a severe growth delay in order to prevent fasting hypoglycemia. Frequent, small meals, restricted in glucose and galactose, and raw cornstarch administration at night are used to prevent metabolic acidosis, which may occur at times of surgery or other stresses. Hypercholesterolemia may require a medical treatment with statins after five years of age; bicarbonate supplementation may be required to balance the urinary bicarbonate loss [63].

According to the available data, universally accepted guidelines for the management of these types of GSDs have not been defined. Nevertheless, an appropriate follow-up should be provided, in order to establish a good metabolic control and monitor the possible complications. Medical and nutritional evaluations and blood assessment, including complete liver and renal function, lipid profile, calcium-phosphate metabolism, serum electrolytes, blood gas analysis and urinalysis, should be fulfilled every 6 months on average; a higher frequency is recommended in younger patients and in those who have not achieved a metabolic balance. A continuous glucose monitoring may be helpful to survey the glycemic fluctuations, especially in younger patients. Alpha-fetoprotein levels along with abdomen ultrasound can be used to screen for hepatocellular carcinoma, even though there are no validated surveillance protocols to date [37]. Liver fibroscan might be an useful and non-invasive tool for the monitoring of the progression of fibrosis/cirrhosis in GSD type IV, VI and IX [64, 87].

GSD type IV patients require a complete cardiac function evaluation, including electrocardiogram and echocardiography. For patients with GSD types VI and IX after 5 years of age a cardiac evaluation is recommended every 1–2 years [44].

Regarding the bone metabolism, a careful assessment of calcium and vitamin D intake and monitoring of 25-OH vitamin D level is recommended. Calcium, phosphate and vitamin D supplementations, along with annual DXA scan evaluation, are required to prevent osteopenia and fractures, particularly in GSD type XI, along with a surveillance of renal function [61]. Skeletal X-Rays are required in GSD type XI to evaluate rickets evolution [55, 56]. Recommendations for vitamin and mineral supplementation are based on individual patient’s diet and nutrient needs.

## Conclusions

GSDs type 0, IV, VI, IX and XI with liver involvement may have a similar clinical presentation. However, these diseases exhibit a phenotypic continuum, and even in the mildest forms, regular monitoring and dietary adjustments are necessary to restrain disease progression and complications. Some cases may exhibit a clinical burden with severe organ complications. Building a proper knowledge among physicians about these rare conditions is crucial to improve prognosis and quality of life of patients, especially those affected by the most severe forms. Further studies are needed to outline the genotype–phenotype correlation and define personalized therapies and management.

## Data Availability

Not applicable.

## Abbreviations

ChREBP: Carbohydrate-response element-binding protein
GBE: Glycogen branching enzyme
GLUT2: Glucose transporter 2
GSD: Glycogen storage disease
GYS: Glycogen synthase
HNF4α: Hepatocyte nuclear factor 4α
PYGL: Glycogen phosphorylase
OGTT: Oral glucose tolerance test
PhK: Phosphorylase kinase
SLC2A2: Solute carrier family member 2
